# Improving the Antioxidant Activity and Flavor of Faba (*Vicia faba* L.) Leaves by Domestic Cooking Methods

**DOI:** 10.3390/antiox11050931

**Published:** 2022-05-09

**Authors:** Shucheng Duan, Soon-Jae Kwon, Chan Saem Gil, Seok Hyun Eom

**Affiliations:** 1 Department of Smart Farm Science, College of Life Sciences, Kyung Hee University, Yongin 17104, Korea; dsc97@khu.ac.kr (S.D.); cskil@khu.ac.kr (C.S.G.); 2 Advanced Radiation Technology Institute, Korea Atomic Energy Research Institute, Jeongeup 56212, Korea; soonjaekwon@kaeri.re.kr

**Keywords:** domestic cooking, phytochemicals, antioxidants, volatiles, harmful compounds

## Abstract

Faba leaves are an unusual vegetable which contain not only a range of functional phytochemicals, but also certain undesirable flavors, which limit their consumption. In this study, several cooking methods (microwaving, roasting, steaming, and boiling), which are expected to reduce the odd flavors, were evaluated in terms of both health benefit effects and odd flavor factors, including antioxidant activities and the content of non-volatile and volatile organic compounds (VOCs). A cooking time of 5 min was selected because of the high content of *l*-dopa (*l*-3,4-dihydroxyphenylalanine) and aim of reducing the undesirable flavors of the cooked faba leaves. Microwaving and steaming significantly increased the *l*-dopa content by 24% and 19%, respectively. Roasting specifically increased the content of flavonols, exhibiting a 28% increase of kaempferol-3-*O*-arabinoside-7-*O*-rhamnoside, representatively, whereas boiling decreased about 50% of most phytochemicals evaluated. Microwaving and steaming treatments significantly increased the antioxidant activities. The *l*-dopa content and antioxidant activities of the processed faba leaves were strongly positively correlated with either an *R*^2^ = 0.863 of DPPH radical scavenging activity or an *R*^2^ = 0.856 value of ABTS radical scavenging activity, showing that *l*-dopa was a key antioxidant. All cooking methods potentially improved the flavor of the cooked faba leaves compared with that of the fresh leaves, because they significantly reduced the contents of VOCs such as alcohols, aldehydes, and ketones. These VOCs were the main components (>90%) in the fresh leaves. Adverse aromatic hydrocarbons were newly formed by the microwaving treatment, typically producing *p*-xylene, which is known to be a harmful dose-dependent compound, but they were not detected in leaves processed by the other cooking methods; therefore, although microwaving efficiently increased antioxidant activity, the chemical safety of the aromatic hydrocarbons produced need further study.

## 1. Introduction

Faba leaves form about 12.7% to 27.1% of by-products arising from faba bean (*Vicia faba* L.) cultivation in terms of the dependence of cultivars [1]. This by-product is usually discarded as waste in most countries where faba beans are grown; however, faba leaves are regarded as an unconventional but valuable vegetable in some regions of China and Italy, eaten either fresh or after domestic cooking. Recently, several studies have demonstrated that the faba leaf is an excellent source of nutrition, not only because of its proximate composition, principal cations, and sugars, but also due to its high contents of phenolics and flavonoids [2,3].

**Figure 1:**
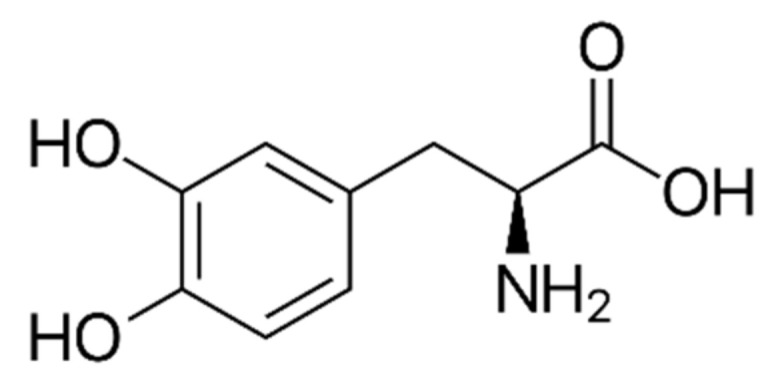
The structure of *l*-dopa.

*l*-dopa (Figure 1), a representative phenolic acid, accumulates in faba leaves at relatively high levels compared with other crops containing *l*-dopa [4,5], and has been reported to improve Parkinson’s disease symptoms [6]. Our recent study has suggested that *l*-dopa also possesses strong antioxidant activity (radical scavenging ability), one of the key antioxidant contributors in faba leaves [3]; however, the chemical synthesis of *l*-dopa is time-consuming and expensive, and involves the use of toxic compounds [7]. Consuming natural *l*-dopa from enriched plant resources such as faba leaves has thus become important as far as health benefits are concerned.

Faba leaves are also rich sources of flavonoids. Neugart et al. [8] have reported that kaempferol is the main aglycone of the various types of flavonol, and it forms various glycosides in different cultivars of faba leaves. A study by Duan et al. [3] reported that the total flavonoid content of the faba leaf varies with its maturity, with young leaves (66.96 mg of catechin equivalent/g dry weight) possessing more than 1.5 times as many flavonoids as old leaves. The health benefits of flavonoids regarding antioxidant, anti-inflammatory, anti-diabetic, and other health-promoting properties have been widely reported [9,10].

Although faba leaves are excellent sources of phytochemicals with various bioactivities, their consumption is often limited because of the presence of undesirable flavors. The volatile organic compounds (VOCs) in faba leaves are considered to be responsible for their grassy smell, which has been widely reported as being related to the plant’s defense system [11,12]. No studies seem to have identified the major contributors causing this unattractive grassy smell among the various VOCs, or how to remove them and thereby promote the better use of this agricultural waste in people’s daily diets.

As mentioned above, faba leaves are consumed either fresh or after domestic cooking. Several studies have reported on the effects of domestic cooking on the variation in composition and bioactivity of leafy vegetables, concluding that they are significantly affected not only by the cooking methods and degree of processing, but also by the plant species and cultivars [13,14,15]. It is also important to point out how domestic cooking methods can improve the flavor of vegetables. Jiang et al. [16] reported that thermal pre-treatments of faba beans significantly improved their flavor by removing the “beany flavor”. Other studies investigating the effect of cooking on changes in VOCs of vegetables such as broccoli, cauliflower, radishes and mushrooms, have also reported significant success in removing undesirable aromas and enhancing attractive flavors [17,18,19]; however, several factors need to be considered for producing faba leaves of high quality, in terms of selecting appropriate cooking methods and the degree of cooking, to provide a high content of bioactive components and to improve their flavor.

Therefore, the aim of this study was to investigate the effects of four commonly used domestic cooking methods—microwaving, roasting, steaming, and boiling—focusing on antioxidant activities and the contents of volatile compounds for flavor improvement. The changes in *l*-dopa content and flavors were determined according to the processing degree (time) of each domestic cooking method. The samples obtained under optimal conditions were analyzed for flavonoid metabolites, total phenolic content (TPC), total flavonoid content (TFC), antioxidant activities (radical scavenging ability), and VOCs.

## 2. Materials and Methods

### 2.1. Chemicals and Reagents

The *l*-dopa and kaempferol-3-glucoside standards were purchased from the Sigma Aldrich Co. (St. Louis, MO, USA) and the HWI group (Rülzheim, Germany), respectively. Other chemicals and solvents used were of analytical grade.

### 2.2. Plant Materials and Domestic Cooking

Faba leaves (Accession number: PI 577722) were obtained from the greenhouse of Kyung Hee University (37° 14′ 36.0″ N 127° 04′ 52.6″ E, Yongin, Korea) in October, 2021. The seeds were planted in August, 2021, using a previously reported method [4]. Intact and undamaged old leaves collected from the base of the plant were washed in distilled water, then wiped with gauze, and were used for the experimental treatments and analysis.

Twenty grams of fresh leaves were used for each processing treatment. For the control group (freeze-dried (FD)), fresh leaves were lyophilized at −80 °C using a freeze dryer (IlshinBioBase, Dongducheon, Korea). Four kinds of domestic cooking methods were applied to evaluate faba leaf characteristics induced by either dry (microwave and roasting) or wet (steaming and boiling) heating. For microwaving (MW), the faba leaves were placed in a glass dish then heated in a commercial microwave (RE-C20YW, Samsung Electronics Co., Ltd., Suwon, Korea) at 700 W for 1, 5, 10, or 12 min. The cooking times were based on preliminary test results showing that the leaves were burned if the time exceeded 12 min. For roasting (RT), the leaves were roasted at 200 ± 20 °C in a commercial cooking pot for 1, 5, 10, or 15 min. The pot surface temperature was monitored using a hand-held infrared thermometer (DT8380, Tianjin Cheerman Technology Co., Ltd., Tianjin, China). For steaming (SM), the leaves were treated following a previously described method [3]. One liter of water was placed in a pot and heated. During the boiling process, fresh leaves were placed on the tray and then immediately covered by a lid. The steaming time was set at 1, 5, 10, or 15 min. For boiling (BL), the leaves were placed in boiling water (1.5 L) for 1, 5, 10, or 15 min. After these different cooking methods, the cooked faba leaves were quickly cooled using ice packs and the surface water was removed using gauze. The samples were then lyophilized in a freeze dryer until totally dry then ground to a powder using a mortar and sieved through a 100-mesh. All the treatments were performed three times. The remaining fresh leaves were stored at −18 °C for further analysis.

### 2.3. Sample Extraction

The samples were extracted as described by Duan et al. [3] with some modifications. Fifty milligrams of the powdered dried samples were mixed with 1.5 mL of 50% aqueous methanol for 60 min with sonication at a temperature below 40 °C. The supernatants were gathered after centrifugation at 14,240× *g* for 15 min, then were stored at −18 °C for further analysis.

### 2.4. High Performance Liquid Chromatography (HPLC) Analysis of Antioxidant Phytochemicals

#### 2.4.1. HPLC Analysis of *l*-dopa

The *l*-dopa content was determined as described previously [3]. Briefly, 100 µL of the supernatant were diluted with 900 µL of 50% aqueous methanol. After filtration through a 0.45-µm membrane filter, the samples were analyzed by gradient elution on a C18 column (Prontosil 120-5-C18-SH 5.0 μm (250 × 4.6 mm), Bischoff, Leonberg, Germany) with an HPLC (Waters 2695 Alliance, Waters Inc., Milford, MA, USA). Solvent A was 0.3% formic acid in water (*v*/*v*) and solvent B was 0.3% formic acid acetonitrile. The following gradient was used for solvent B: 2% (0–9 min), 2–80% (9–10 min), 80% (10–14 min), 80–2% (14–16 min), and 2% (16–20 min). An injection volume of 5 µL and a flow rate of 0.8 mL/min were used. The peak of *l*-dopa was detected at 280 nm by a Waters 996 photodiode array detector (Waters Inc.).

#### 2.4.2. HPLC and LC-MS/MS (Mass Spectrometry) Analysis of Flavonoids

The flavonoids were analyzed as described by Neugart et al. [8] with some modifications. The sample, 100 µL of the supernatant obtained in 2.3, was diluted with 900 µL of 50% aqueous methanol. After filtration through a 0.45-µm membrane filter, the flavonoids were separated by gradient elution on a C18 column (Kinetex 100 Å, 5 µm (150 × 4.6 mm), Phenomenex, Torrance, CA, USA) with a Waters 2695 Alliance HPLC (Waters Inc., Milford, MA, USA). Solvent A was 5% acetic acid in water (*v*/*v*) and solvent B was acetonitrile. The following gradient was used for solvent B: 5–14% (0–3 min), 14–16% (3–30 min), 16% (30–40 min), 16–90% (40–42 min), 90% (42–44 min), 90–5% (44–46 min), and 5% (46–49 min). An injection volume of 5 µL and a flow rate of 0.5 mL/min were used. The peaks of the flavonoids were detected at 370 nm by a Waters 996 photodiode array detector (Waters Inc.). The quantitative analysis of flavonoids was calculated by the standard compound kaempferol-3-glucoside.

The compounds were identified on an LC-MS/MS instrument (Thermo-Finnigan LTQ-Orbitrap, Thermo Scientific, Waltham, MA, USA). The HPLC conditions were the same as mentioned above. Xcalibur software was used for data acquisition. The MS/MS analytical conditions were as follows: the negative ion mass spectra of the column elute were recorded in the range *m/z* 150–2000; the fragmentation and capillary voltages were 0.2 kV and 4.5 kV, respectively; the capillary temperature was kept at 300 °C at 4.5 kV; the collision energy was set at 35 eV; and the sheath gas (nitrogen) flow rate was maintained at 10 L/min.

### 2.5. Determination of Total Phenolics Content (TPC), Total Flavonoids Content (TFC), and Antioxidant Activities

The sample consisted of 50 µL of the supernatant obtained in 2.3 diluted with 950 µL of 50% aqueous methanol. Colorimetric assays were used to determine TPC and TFC as described by Lim and Eom [20]. The TPC of the faba leaves was expressed as mg of gallic acid equivalent (GAE)/g dry weight (d.w.) and the TFC as mg of catechin equivalent (CE)/g d.w. To determine the antioxidant activity, the DPPH and ABTS free radical scavenging abilities were measured as described by Lim et al. [21] The radical scavenging abilities were expressed as mg of vitamin C equivalent (VCE)/g d.w.

### 2.6. Headspace Solid-Phase Microextraction Gas Chromatography–Mass Spectrometry (HS-SPME-GC–MS) Analysis of VOCs

#### 2.6.1. Sample Preparation

Batches of faba leaves stored at −18 °C were allowed 2 min to recover to room temperature, then immediately treated for 5 min according to the domestic cooking methods described in Section 2.2. Any excess water was removed using gauze. The recovered but uncooked faba leaves were used as the control group (Fresh). The obtained samples were immediately cooled in liquid nitrogen then ground to a fine powder using a mortar. The faba leaf powder was transferred into a plastic tube, sealed, then stored at −80 °C until analysis.

#### 2.6.2. HS-SPME and GC–MS Analysis

The sample (1.5 g) was immediately put into a headspace bottle, then 1 g of sodium chloride was added. The volatile compounds were extracted by the HS-SPME method, using a PDMS/DVB (polymethyl siloxane/divinyl benzene) fiber (65 µm, 23 Ga (pink), Supelco, Bellefonte, PA, USA). The temperature was equilibrated at 60 °C, then the extraction continued for 30 min. The fiber was desorbed into the injection port of a GC–MS (GC: Trace1310; MS: Triple quadrupole mass spectrometer (TSQ 8000), Thermo Fisher Scientific Inc., Waltham, MA, USA) at 230 °C for 2 min. The ramp temperature in the GC oven was maintained at 40 °C for 5 min, increased to 120 °C at 8 °C /min, increased to 160 °C at 2 °C /min, increased finally to 240 °C at 4 °C /min, then held there for a further 10 min. The MS was operated in scan mode at 70 eV with the electron ionization (EI) source kept at 250 °C. The mass acquisition range was set at 35–550 *m/z*. The VOCs were identified using the NIST mass spectral search program with the NIST/EPA/NIH Mass Spectral Library (ver. 2.0). The volatiles were quantified as the percentage for each volatile of the total peak area of the fresh sample.

### 2.7. Statistics Analysis

The data were analyzed statistically using SAS software (Enterprise Guide 7.1 version; SAS Institute Inc., Cary, NC, USA). Significant differences between experimental groups were evaluated using ANOVA followed by Tukey’s HSD test at a level of *p* < 0.05.

## 3. Results and Discussion

### 3.1. Effects of Domestic Cooking on Faba Leaf l-dopa Content Variations

**Figure 2:**
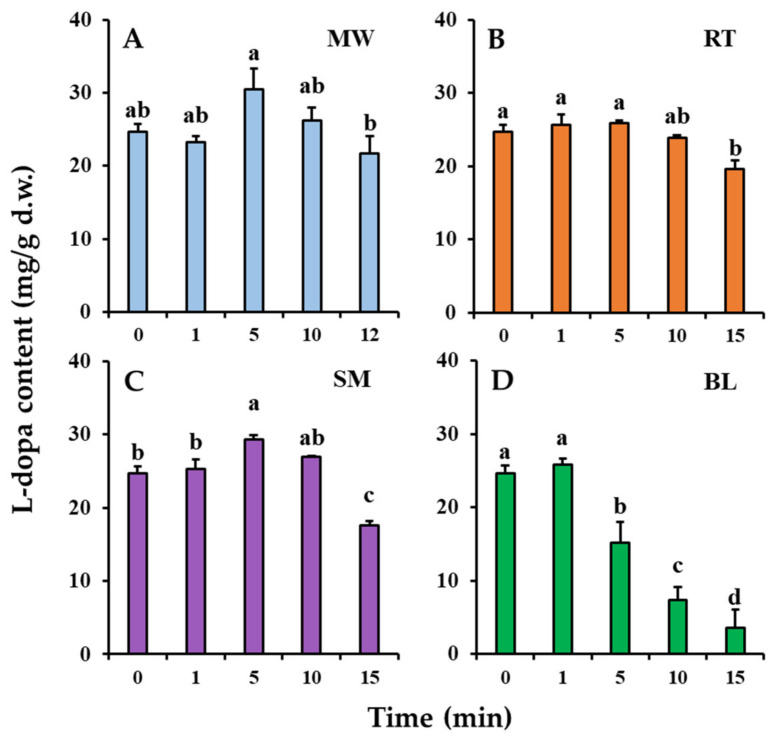
Changes in *l*-dopa content of faba leaves with cooking time during: (**A**). microwaving (MW); (**B**). roasting (RT); and (**C**). steaming (SM); and (**D**). boiling (BL). Values are averages with standard errors from triplicate experiments. Different letters on the graph bars indicate a significant difference at *p* < 0.05 (Tukey’s HSD test).

Changes in the *l*-dopa content of the faba leaves during the domestic cooking treatments are shown in Figure 2. The *l*-dopa content of the uncooked (0 min) faba leaf of 24.69 mg/g d.w. changed significantly after domestic cooking. After 5 min of microwaving treatment, the *l*-dopa content increased to 30.51 mg/g d.w. (Figure 2A) but then began to decrease as the cooking time increased. For roasting, there were no significant changes in the *l*-dopa content after 1 and 5 min (Figure 2B), but a significant decrease to 19.57 mg/g d.w. was observed after 15 min of treatment, to a similar level as that after microwaving for 12 min (21.71 mg/g d.w.). Steaming and boiling are both wet heating methods, but they exhibited different effects on changes in the *l*-dopa content during processing (Figure 2C,D). The longer steaming treatment (15 min) significantly decreased the *l*-dopa content (17.53 mg/g d.w.), but it was increased (29.35 mg/g d.w.) by a relatively short cooking time (5 min); however, a boiling treatment of only 1 min maintained the *l*-dopa content (25.83 mg/g d.w.), but the content decreased significantly as the boiling time increased further.

Faba leaves have been reported to be an excellent natural source of *l*-dopa [4]; however, how to consume *l*-dopa from faba leaves in one’s daily diet is still unclear. The results showed that both microwaving and steaming treatments can efficiently improve the *l*-dopa content after 5 min of the cooking treatment. Interestingly, even though roasting is also a type of dry heating method, there was no effect on improving the *l*-dopa content. Raigar et al. [22] have investigated the effect of microwave roasting on changes in the physicochemical properties of peanuts, and found that the larger globules of starch and proteins in the peanuts disintegrated as the microwave power and time increased based on SEM. The reason why microwaving increased the *l*-dopa content could be explained by the fact that that the microwave treatment can help create micropores on the faba leaf, thus accelerating the release of relatively low molecular weight compounds such as *l*-dopa. Microwave energy can also directly penetrate the food matrix, providing faster heating than conventional methods such as roasting, based on surface heating [23]. Our previous results suggested that steaming significantly decreased the *l*-dopa content of faba leaves after 15 min of treatment [3], which was consistent with the results of the present study; however, the pattern of *l*-dopa content first increasing then decreasing was shown in faba leaves during steam processing times of up to 15 min (Figure 2C). The longer steaming treatment could have damaged the *l*-dopa structure, whereas the short-time treatment had increased the extraction efficiency. Of the four domestic cooking methods, the most significant reduction in *l*-dopa content was observed with the boiling treatment (Figure 2D). Wet heating has been found to cause a stronger deterioration of *l*-dopa than dry heating. The significant reduction in *l*-dopa after boiling was caused either by its structural breakdown or by the release of water [13]; therefore, regarding *l*-dopa consumption, faba leaves should be consumed either fresh, or with 5 min of domestic cooking using methods such as microwaving, roasting, and steaming, or with 1 min of boiling treatment; however, as previously mentioned, the consumption of the faba leaf is also hampered by the presence of undesirable flavors. Regarding this aspect, the flavor of all the samples treated for 5 min had improved properties compared with samples processed for a short time; therefore, a treatment time of 5 min is recommended for these four domestic methods when cooking faba leaves, to obtain as much *l*-dopa as possible, as well as a better flavor. The analysis of the flavonoid metabolites, TPC, TFC, antioxidant activities, and VOCs were thus performed on samples treated for 5 min.

### 3.2. Effects of Domestic Cooking on Faba Leaf Flavonoid Metabolites Variations

The only flavonoids detected in faba leaves were flavonol glycosides (Table 1), which is consistent with results reported by Neugart et al. [8] and Yan et al. [24] Ten types of kaempferol glycoside and three types of quercetin glycoside were identified among the flavonol glycosides.

The effect of domestic cooking on changes in the content of each flavonol metabolite in faba leaves is shown in Table 2. For the content of each kaempferol glycoside in the faba leaves, there were no significant differences between the freeze-dried (control), microwaving, and steaming treatments. The roasting treatment specifically increased the contents of K-3-ara-7-rha (6.13 mg/g d.w.) and K-3-rha-gal (glu)-7-rha (1.21 mg/g d.w.), but had no effect on the other metabolites. The boiling treatment significantly reduced the content of the faba leaf kaempferol glycosides, particularly of K-3-ara-7-rha (2.83 mg/g d.w.), K-3-rha-7-rha + un (1.56 mg/g d.w.), and K-3-acetyl-rha-gal-7-rha (0.29 mg/g d.w.). Otherwise, the effects of the four domestic cooking methods on the contents of quercetin glycosides were significantly different from those of the kaempferol derivatives. Compared with fresh leaves, leaves cooked by microwaving and steaming both exhibited higher contents of Q-3-rha-gal (glu)-7-rha and Q-3-rha-ara-7-rha; however, there were no significant variations in Q-3-rha-glu content, whichever treatment was used.

Kaempferol glycosides are the main type of flavonol compound in faba leaves [24,25]; however, the variations in the content of individual compounds with each cooking method were significantly different. Similar results have also been observed by Wu et al. [26] who investigated the changes in the flavonoid contents of broccoli caused by three domestic cooking methods. Quercetin derivatives only formed a small proportion of flavonols in faba leaves (Table 2); however, all of the cooked faba leaves exhibited a higher content of quercetin derivatives than the fresh leaves. These differences in the pattern of variation between kaempferol and quercetin glycosides might be explained by the variation in the thermal stability of the compounds caused either by the type of aglycone or the variation in the glycosides attached to the aglycone [27]. It has often been suggested that the changes in the content of individual polyphenols during domestic cooking are mainly affected by an increase in the efficiency of extracting the compounds, and by a reduction in the content of compounds caused by thermal degradation or water leaching [13,15].

### 3.3. Effects of Domestic Cooking on Changes in the TPC, TFC and Antioxidant Activities of Faba Leaves

**Figure 3:**
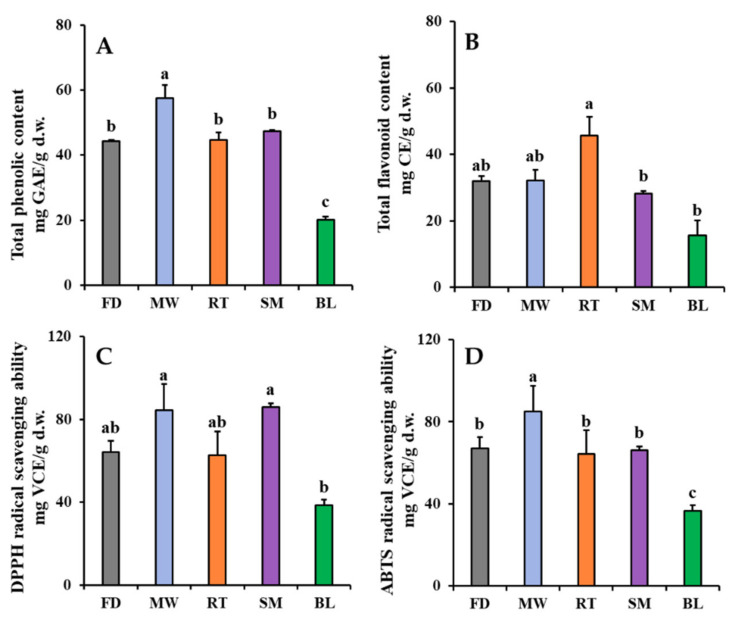
Changes in TPC (**A**), TFC (**B**), DPPH radical scavenging ability (**C**), and ABTS radical scavenging ability (**D**) of faba leaves after treatment by four domestic cooking methods (FD: freeze-dried (Control); MW: microwaving; RT: roasting; SM: steaming; BL: boiling). Values are averages with standard errors from triplicate experiments. Different letters on the graph bars indicate significant differences at *p* < 0.05 (Tukey’s HSD test).

The effects of domestic cooking on changes in the TPC, TFC, and antioxidant activities of faba leaves are shown in Figure 3. The TPC was 44.26 mg GAE/g d.w. in freeze-dried faba leaves (Figure 3A). For the cooked leaves, only the microwaving treatment significantly increased TPC (57.56 mg GAE/g d.w.) compared with the other treatments. The increased *l*-dopa content after the microwaving treatment was probably responsible for this result (Figure 2A); however, there were no significant differences in TPC between the steamed and freeze-dried faba leaves, with even a short steaming time also increasing the *l*-dopa content (Figure 2B). This might be explained by a reduction in the polyphenol compounds that are relatively sensitive to steaming [28]. The roasting treatment maintained the level of TPC, but the boiling treatment decreased it (Figure 3A). These results were consistent with those observed in the changes in *l*-dopa content (Figure 2B,D). Overall, a high positive correlation (0.958) was observed between the *l*-dopa content and TPC (Table S1). The roasting treatment increased the TFC of faba leaves (45.73 mg CE/g d.w.) compared with freeze drying (32.02 mg CE/g d.w.), whereas the other cooking methods did not (Figure 3B). This result was consistent with the changes in the contents of flavonoid metabolites (Section 3.2).

The variation in the DPPH/ABTS radical scavenging abilities of the freeze-dried and cooked faba leaves are shown in Figure 3C,D, respectively. Briefly, similar patterns in both measurements of antioxidant activity of the faba leaves were observed: they were increased by the microwaving treatment, not significantly changed by the roasting treatment, maintained by the boiling treatment, and the steaming treatment significantly increased the DPPH radical scavenging ability but not the ABTS radical scavenging ability. One explanation could be that even though the *l*-dopa content was increased by steaming, there was no significant change in the TPC (Figure 2C and Figure 3A). High levels of *l*-dopa, vitamin C, TP, and TF have been reported to be responsible for strong antioxidant activity in dry heated and wet heated faba leaves [3]; however, in the present study, the correlation analysis suggested that the radical scavenging activities of fresh and cooked faba leaves were mainly caused by the *l*-dopa content (0.863 with DPPH radical scavenging activity; 0.856 with ABTS radical scavenging activity) and TPC (0.854 with DPPH radical scavenging activity; 0.979 with ABTS radical scavenging activity), not by the individual flavonol and TFC content (Table S1). *l*-dopa has been reported to be an effective antioxidant in several in vitro assays such as radical scavenging, reductive ability and anti-lipid peroxide [29]. Even flavonol glycosides have been reported to also contribute strong antioxidant activities in numerous plants, and the activities are significantly varied depending on flavonol types and contents [30]. *l*-dopa is a predominant compound that exists in faba leaves among the polyphenols detected in this study (Figure 2 and Table 2). Our previous study has suggested that *l*-dopa is more powerful than vitamin C in terms of radical scavenging abilities [3]. Moreover, a high correlation was observed between the DPPH radical scavenging ability and *l*-dopa content, compared with that found between the ABTS radical scavenging ability and TPC. These findings can be supported by a previous study where the same concentration of an *l*-dopa standard compound (0 to 20 mM) exhibited a stronger DPPH radical scavenging ability than the ABTS radical scavenging ability [3]. Here, it is important to point out the different mechanisms for evaluating antioxidant action between DPPH and ABTS assays [31,32]. In a DPPH assay, DPPH is a specialized lipophilic radical, which can be neutralized into the reduced forms of either DPPH-H (by receiving a hydrogen atom) or DPPH (by accepting an electron); however, the ABTS assay is suitable when evaluating antioxidant components which have a hydrophilic or lipophilic character. The assay is only based on a hydrogen atom transfer reaction. Additionally, the important parameter when evaluating the antioxidant action of a single electron transfer is ionization potential, whereas that of hydrogen atom abstraction is bond dissociation enthalpy.

### 3.4. Effects of Domestic Cooking on Changes in the Contents of VOCs of Faba Leaves

**Figure 4:**
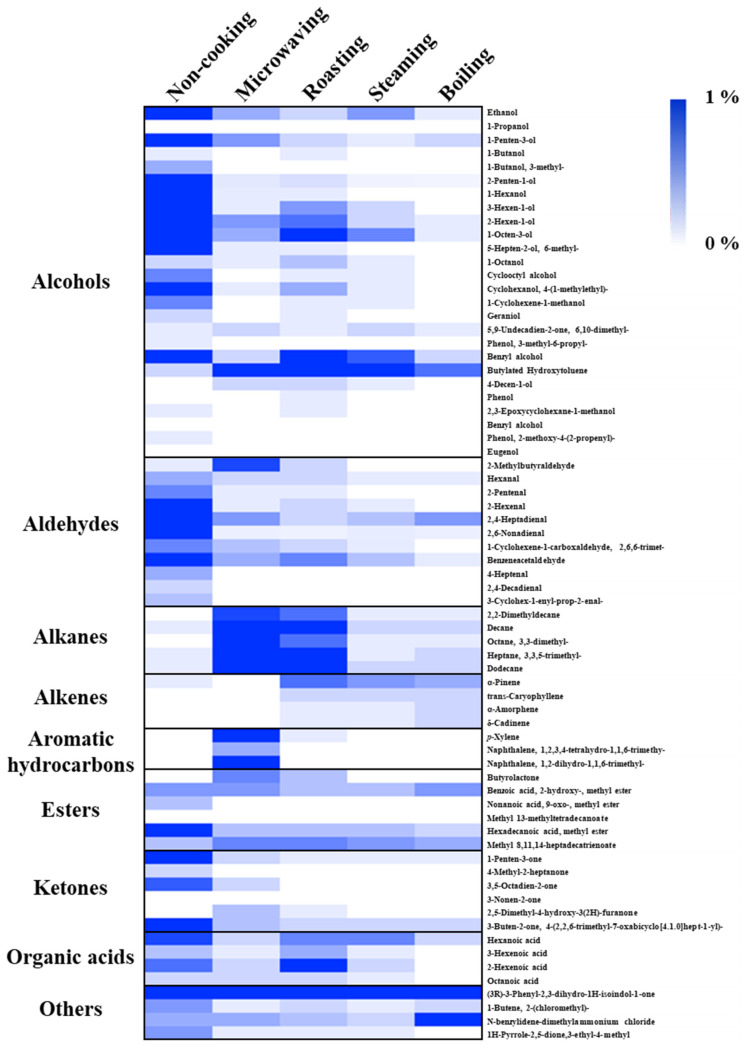
Heat map of VOCs detected in cooked faba leaves by HS-SPME-GC–MS. The data are presented as the percentage for each volatile compound (peak area) of the total peak area of fresh faba leaves.

**Figure 5:**
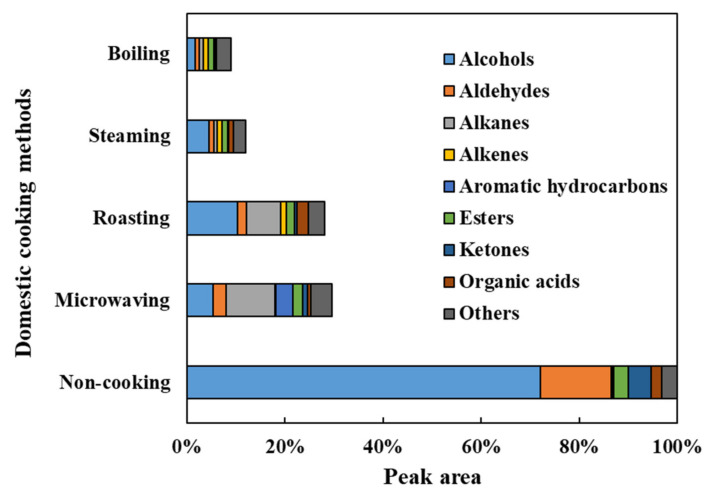
The profile of nine groups of VOCs detected in fresh and cooked faba leaves. Values of groups are the totals within each compound content.

The VOCs in the faba leaves were analyzed by HS-SPME-GC/MS. Overall, 69 compounds were detected (Figure 4 and Table S2). The data are shown as the percentage for each volatile based on the total peak area of the uncooked faba leaves. These compounds were categorized into 9 groups: 26 alcohols, 11 aldehydes, 5 alkanes, 4 alkenes, 3 aromatic hydrocarbons, 6 esters, 6 ketones, 4 organic acids, and 4 other compounds (Figure 5, Table S2). The domestic cooking methods significantly reduced the total content of VOCs, by 29.52%, 28.11%, 11.88%, and 9.04%, after microwaving, roasting, steaming, and boiling, respectively (Figure 5). Similar results have also been reported by Selli et al. [19] who found that boiling and oven cooking significantly reduced the content of aroma compounds in two mushroom cultivars.

#### 3.4.1. Alcohols

Alcohols (72.02%), the representative VOCs in fresh faba leaves, were significantly reduced by domestic cooking: by 10.37% for roasting, by 5.24% for microwaving, by 4.50% for steaming, and by 1.65% for boiling (Figure 5). Of the alcohols (Figure 4, Table S2), 1-octen-3-ol (23.55%), 1-penten-3-ol (13.43%), 3-hexen-1-ol (9.10%), 2-penten-1-ol (7.29%), 2-hexen-1-ol (5.54%), benzyl alcohol (3.15%), 1-hexanol (2.52%), and ethanol (2.11%) contributed more than 90% of the total alcohols content, which are compounds that are widely reported to contribute to the faba leaf aroma [33,34,35]. Moreover, 1-octen-3-ol, which is known to contribute to the smell of mushrooms, has been reported as a key volatile compound in faba flours [35]. Additionally, 1-penten-3-ol, 3-hexen-1-ol, 2-penten-1-ol, and 2-hexen-1-ol have been suggested as being pungent, as well as possessing the flavors of grass, greens, leaves, and so on [36], which are traits commonly known to unfavorable volatiles. The removal of these VOCs by domestic cooking can be considered to be an important method for improving the flavor of faba leaves. In particular, the content of a synthetic compound, butylated hydroxytoluene (BHT), detected in our analysis (Figure 4, Table S2), increased in the cooked samples of faba leaves. It is therefore important to point out the two sides of the HS-SPME technology: its convenience for analyzing VOCs, in addition to its potential for contaminating samples caused by the fiber coatings [37].

#### 3.4.2. Aldehydes/Ketones/Esters

Of the nine groups of VOCs in the fresh faba leaves, aldehydes (14.44%), ketones (4.78%), and esters (2.89%) were ranked second, third, and fifth regarding peak areas, respectively (Figure 5, Table S2). For aldehydes, 2,4-heptadienal (4.18%), benzene acetaldehyde (3.77%), 2,6-nonadienal (E, Z) (2.08%), and 2-hexenal (E) (1.91%) contributed more than 80% of the total contents. Among the represented aldehydes, several components have been known to contribute to various smells, exhibiting 2,4-heptadienal with the odorant of nuts and fats, benzene acetaldehyde with the flavor of hawthorn, and 2-hexenal (E) with the smell of apples and fresh green leaves [36]. Moreover, 1-Penten-3-one is associated with fish and pungent smells, and hexadecanoic acid methyl ester is associated with fatty, oily, and waxy flavors, which form approximately 50% of the detected VOCs that are categorized as ketones and esters, respectively (Table S2) [36]. After domestic cooking, the amount in each group decreased, with a similar pattern being observed in that boiling caused the most significant reduction, followed by steaming, roasting, then microwaving (Figure 4, Table S2).

#### 3.4.3. Alkanes/Alkenes/Aromatic Hydrocarbons

In the fresh faba leaves, the alkanes, alkenes, and aromatic hydrocarbons were three groups present, but only in small amounts of 0.37%, 0.15%, and 0.03%, respectively (Figure 5, Table S2). The content of each group was increased similarly by domestic cooking with a different pattern according to the method. For the alkanes, faba leaves treated by microwaving exhibited 10.03% of the total peak area of fresh faba leaves, followed by 6.95% for roasting, 0.79% for boiling, and 0.71% for steaming. Of these alkanes, the significant increase in the contents of decane, dodecane, and 3,3,5-trimethyl-heptane were responsible for the increase of the total alkane content (Figure 4, Table S2). Similar results were observed by Yang et al. [38] who reported that decane and dodecane increased during the drying of the edible mushroom, *Flammulina velutipes*, but were not detected in the fresh fungus. Interestingly, unlike the alkanes, the alkene content was increased by the roasting (1.16%), steaming (0.91%), and boiling (0.99%) treatments, but not by the microwaving (0.16%) treatment. This might be explained by the difference in the heating mechanism between the microwave treatment and traditional heat transfer methods, resulting in different modes of transforming VOCs during processing [23]. It is important to note the effect of microwaving on changing the contents of aromatic hydrocarbons (3.56%): it increases the content of *p*-xylene (1.61%) by 54 times, as well as newly synthesized naphthalene derivatives (1.94%) (Figure 4, Table S2). *p*-Xylene is an important aromatic hydrocarbon found in faba beans and sprouts [35]; however, unlike its relatively non-toxic isomers (*o*-xylene and *m*-xylene), exposure to *p*-xylene vapor at over one hundred parts per million has been suggested to cause toxic effects such as irritation to the eyes and throat, possible chest tightening, and an abnormal gait [39]. Although Selli et al. [19] have reported that both boiling and oven cooking increased the *m*-xylene content of two different cultivars of mushroom, the reason why *p*-xylene was specifically boosted by microwaving in the present study is still unclear. Microwaves are electromagnetic radiations, which lie between radio waves and infrared radiations [40,41,42]. With the difference of conventional heating methods, several specific microwave effects caused by the dielectric heating mechanism have been reported, such as superheating, selective heating, exciting water molecules by a particular frequency of microwaves, breaking covalent bonds of polymerized compounds, and so on [23,40,43]; therefore, future studies could investigate how factors of time and power during microwave treatment affect the synthesis of the relatively harmful *p*-xylene, not only in faba leaves, but also in other food matrices. In addition, future studies could also investigate whether the increased *p*-xylene content is harmful to human health.

#### 3.4.4. Organic Acids/Other Compounds

Four types of organic acid (2.01%) were detected in the fresh faba leaves (Table S2). Except for roasting (2.26%), microwaving (0.65%), steaming (0.91%), and boiling (0.22%), treatments significantly reduced the contents of organic acids (Figure 4 and Figure 5). Four compounds were also detected in the faba leaves and categorized as others, but their contents were not affected by domestic cooking as the other groups were (Figure 4 and Table S2).

## 4. Conclusions

To obtain faba leaves with rich nutrition and a good flavor, a cooking time of 5 min is recommended for all the domestic cooking methods investigated in the present study. Compared with fresh faba leaves, those cooked by microwaving and steaming exhibited a significantly increased *l*-dopa content. The content of flavonoids, particularly kaempferol glycosides, increased in leaves after the roasting treatment. Of the cooking methods studied, boiling had the strongest effect on reducing both the non-volatile compounds and VOCs. Alcohols, aldehydes, ketones, and esters formed more than 90% of the VOCs detected in the fresh faba leaves, but their contents were significantly reduced after cooking. In contrast, the content of alkanes increased significantly after the microwaving and roasting treatments. Aromatic hydrocarbons increased, particularly in faba leaves treated by microwaving, significantly increasing the contents of the relatively harmful *p*-xylene and newly synthesized naphthalene derivatives. Importantly, the microwaving and steaming treatments both significantly increased the antioxidant activities of the faba leaves. The *l*-dopa content and antioxidant activities of the processed faba leaves were also strongly positively correlated. Overall, all of the cooking methods tested in this study can be applied to faba leaves to remove undesirable flavors, with microwaving and steaming being recommended to significantly improve the antioxidant activities and *l*-dopa content; however, the use of microwaving may be limited by the significantly increased *p*-xylene content, which may have harmful effects on human health. Further studies should therefore be considered to clarify any negative effects of applying microwave treatment when cooking faba leaves for reasons of food safety. In addition, the sensory evaluation and bioaccessibility analysis of domestically cooked faba leaves should also be performed in further studies.

## Figures and Tables

**Table 1:** Flavonoids identified in faba leaves by LC-MS/MS.

Peak No.	Rt (min)	MW	MS	MS/MS	Identification
1	10.87	432.5	431.5		K-3-*O*-rha
2	11.37	886.7	885.7	739, 449.7	K-3-*O*- rha-glc -7-*O*-rha-4′-rha
3	12.71	610.6	609.6	447.2, 301	Q-3-*O*- rha-glc
4	13.43	756.6	755.6	609, 447.3	Q-3-*O*- rha-gal (glc)-7-*O*-rha
5	13.87	726.5	725.5	579.3, 446.2	Q-3-*O*- rha-ara-7-O-rha
6	15.49	594.8	593.8	447.2, 285	K-3-*O*-gal-7-*O*-rha
7	15.83	740.8	739.8	593.3	K-3-*O*-rha-gal (glc)-7-*O*-rha
8	18.03	710.9	709.9	563.3	K-3-*O*-rha-ara-7-*O*-rha
9	19.25	564.6	563.6	417.2, 285.1	K-3-*O*-ara-7-*O*-rha
10	20.15	782.8	781.8	635.4	K-3-*O*-acetyl-rha-gal-7-*O*-rha
11	27.34	578.8	577.8	431.2, 285.1	K-3-*O*-rha-7-*O*-rha
12	27.79	625.1	624.1	579.2	K-G (Unknown)
13	30.06	636.8	635.8	431.1, 285.1	K-3-*O*-acetyl-gal-7-*O*-rha

Abbreviation: K, kaempferol; Q, quercetin; rha, rhamnoside; rha-glc, rhamnoglucoside; gal, galactoside; rha-gal, rhamnogalactoside; rha-ara, rhamnoarabinoside; K-G, kaempferol glycosides.

**Table 2:** Effect of domestic cooking on faba leaf flavonoid (flavonol) glycosides (mg of kaempferol-3-*O*-glucoside equivalent g ^−1^ d.w.).

Flavonols	Glycosides	Domestic Cooking Methods				
		FD	MW	RT	SM	BL
K-G	3-rha	0.22 ± 0.02 a	0.18 ± 0.01 a	0.22 ± 0.05 a	0.18 ± 0.01 a	0.15 ± 0.01 a
	3- rha-glc-7-rha-4′-rha	0.34 ± 0.03 a	0.29 ± 0.03 a	0.31 ± 0.08 a	0.29 ± 0.03 a	0.22 ± 0.02 a
	3-gal-7-rha	0.39 ± 0.04 a	0.36 ± 0.02 a	0.41 ± 0.04 a	0.36 ± 0.02 a	0.35 ± 0.03 a
	3-rha-gal (glc)-7-rha	0.94 ± 0.07 b	0.89 ± 0.03 b	1.21 ± 0.06 a	0.89 ± 0.03 b	0.72 ± 0.00 b
	3-rha-ara-7-rha	1.78 ± 0.27 a	1.79 ± 0.12 a	1.85 ± 0.38 a	1.79 ± 0.12 a	1.39 ± 0.02 a
	3-ara-7-rha	4.78 ± 0.52 ab	4.40 ± 0.12 b	6.13 ± 0.37 a	4.40 ± 0.12 b	2.83 ± 0.15 c
	3-acetyl-rha-gal-7-rha	0.41 ± 0.03 ab	0.48 ± 0.06 ab	0.63 ± 0.06 a	0.48 ± 0.06 ab	0.29 ± 0.03 b
	3-rha-7-rha + un	3.40 ± 0.24 a	3.18 ± 0.06 a	3.90 ± 0.66 a	3.18 ± 0.06 a	1.56 ± 0.08 b
	3-acetyl-gal-7-rha	0.71 ± 0.10 a	0.83 ± 0.06 a	0.75 ± 0.14 a	0.83 ± 0.06 a	0.51 ± 0.04 a
Q-G	3-rha-glc	0.07 ± 0.00 a	0.09 ± 0.01 a	0.07 ± 0.01 a	0.09 ± 0.01 a	0.06 ± 0.02 a
	3-rha-gal (glc)-7-rha	0.12 ± 0.01 b	0.17 ± 0.01 a	0.15 ± 0.01 ab	0.17 ± 0.01 a	0.15 ± 0.00 ab
	3-rha-ara-7-rha	0.06 ± 0.01 b	0.12 ± 0.01 a	0.07 ± 0.00 b	0.12 ± 0.01 a	0.10 ±0.01 ab

Values are averages with standard errors from triplicate experiments. Different letters within the same line indicate significant differences at *p* < 0.05 (Tukey’s HSD test). Abbreviation: K-G, kaempferol glycosides; Q-G, quercetin glycosides; rha, rhamnoside; rha-glc, rhamnoglucoside; gal, galactoside; rha-gal, rhamnogalactoside; rha-ara, rhamnoarabinoside; un, unknown; FD, freeze dried; MW, microwaving; RT, roasting; SM, steaming; BL, boiling.

## Data Availability

The data presented in this study are available in article and Supplementary Materials.

## Supplementary Materials

The following supporting information can be downloaded at: https://www.mdpi.com/article/10.3390/antiox11050931/s1, Table S1: Correlation coefficients between antioxidant activities and metabolite groups assembled with processed faba samples, Table S2: The volatile flavor profiles of faba leaves before and after domestic cooking.
