# Supplementary material for: Improving the Antioxidant Activity and Flavor of Faba (Vicia faba L.) Leaves by Domestic Cooking Methods
Source: Antioxidants (Basel). 2022 May 9;11(5):931. doi: 10.3390/antiox11050931 (PMC9137704; doi:10.3390/antiox11050931)
Supplement: Supplementary file 1 [file antioxidants-11-00931-s001.zip › antioxidants-1712833-supplementary.pdf]

Table S1. Correlation coefficients between antioxidant activities and metabolite groups assembled with processed faba samples.

		L-dopa	Flavonoids (Peak number)[a]												TPC	TFC	DPPH	ABTS
			1	2	3	4	5	6	7	8	9	10	11+12	13				
**L-dopa**		1																
**Flavonoids (Peak number)**	**1**	0.230ns	1															
	**2**	0.291ns	0.884***	1														
	**3**	0.559*	0.141ns	0.128ns	1													
	**4**	0.189ns	-0.476ns	-0.339ns	0.409ns	1												
	**5**	0.234ns	-0.198ns	-0.181ns	0.509ns	0.625*	1											
	**6**	0.214ns	0.248ns	0.041ns	-0.068ns	-0.378ns	-0.175ns	1										
	**7**	0.365ns	0.655**	0.447ns	0.047ns	-0.241ns	-0.272ns	0.552*	1									
	**8**	0.379ns	0.846***	0.904***	0.306ns	-0.169ns	0.087ns	0.088ns	0.48ns5	1								
	**9**	0.514ns	0.656**	0.494ns	0.048ns	-0.335ns	-0.261ns	0.607*	0.957***	0.496ns	1							
	**10**	0.432ns	0.654**	0.613*	0.205ns	0.101ns	0.044ns	0.102ns	0.809***	0.637*	0.769***	1						
	**11+12**	0.694**	0.491ns	0.378ns	0.174ns	-0.191ns	-0.187ns	0.571*	0.836***	0.349ns	0.924***	0.673**	1					
	**13**	0.626*	0.689**	0.741**	0.559*	0.158ns	0.326ns	0.190ns	0.476ns	0.874***	0.498ns	0.652**	0.498ns	1				
**TPC**		0.958***	0.134ns	0.162ns	0.488ns	0.141ns	0.140ns	0.225ns	0.355ns	0.219ns	0.501ns	0.359ns	0.717**	0.475ns	1			
**TFC**		0.488ns	0.283ns	0.111ns	0.166ns	-0.058ns	-0.308ns	0.280ns	0.736**	0.133ns	0.715**	0.588*	0.768***	0.218ns	0.597*	1		
**DPPH**		0.863***	-0.084ns	-0.055ns	0.388ns	0.257ns	0.381ns	0.299ns	0.201ns	0.023ns	0.367ns	0.214ns	0.613*	0.358ns	0.854***	0.314ns	1	
**ABTS**		0.856***	0.110ns	0.209ns	0.330ns	0.165ns	0.160ns	0.187ns	0.305ns	0.257ns	0.452ns	0.357ns	0.662**	0.487ns	0.919***	0.495ns	0.794***	1

Tukey's HSD test, * indicates $p < 0.05$, ** indicates $p < 0.01$, *** indicates $p < 0.001$, and ns indicates no significance. [a] Peak identification is shown in Table 1.

Table S2. The volatile flavor profiles of faba leaves before and after domestic cooking.

Identification	Rt (min)	*m/z*	Treatment				
			**Fresh**	**Microwave**	**Roasting**	**Steaming**	**Boiling**
**Alcohols**			**72.02%**	**5.24%**	**10.37%**	**4.50%**	**1.65%**
Ethanol	9.18	46	2.11%	0.44%	0.25%	0.53%	0.11%
1-Propanol	11.84	60	0.02%	0.00%	0.00%	0.00%	0.00%
1-Penten-3-ol	14.68	86	13.43%	0.47%	0.21%	0.12%	0.20%
1-Butanol	15.72	74	0.10%	0.04%	0.06%	0.00%	0.00%
1-Butanol, 3-methyl-	15.74	88	0.40%	0.00%	0.00%	0.00%	0.00%
2-Penten-1-ol	18.55	86	7.29%	0.15%	0.11%	0.06%	0.07%
1-Hexanol	19.41	102	2.52%	0.06%	0.13%	0.04%	0.01%
3-Hexen-1-ol	20.47	100	9.10%	0.12%	0.47%	0.20%	0.02%
2-Hexen-1-ol	21.07	100	5.54%	0.53%	0.68%	0.21%	0.12%
1-Octen-3-ol	22.4	128	23.55%	0.38%	2.83%	0.61%	0.06%
5-Hepten-2-ol, 6-methyl-	22.85	128	1.35%	0.05%	0.12%	0.03%	0.00%
1-Octanol	26.43	130	0.23%	0.11%	0.29%	0.10%	0.03%
Cyclooctyl alcohol	28.93	128	0.56%	0.01%	0.11%	0.05%	0.00%
Cyclohexanol, 4-(1-methylethyl)-	30.49	156	1.16%	0.06%	0.39%	0.10%	0.00%
1-Cyclohexene-1-methanol	31.99	112	0.57%	0.01%	0.08%	0.06%	0.02%
Geraniol	39.13	154	0.23%	0.02%	0.14%	0.05%	0.00%
5,9-Undecadien-2-one, 6,10-dimethyl-	39.65	194	0.15%	0.16%	0.12%	0.20%	0.11%
Phenol, 3-methyl-6-propyl-	39.74	150	0.14%	0.03%	0.00%	0.01%	0.00%
Benzyl alcohol	40.62	108	3.15%	0.21%	1.44%	0.76%	0.18%
Butylated hydroxytoluene	41.74	220	0.17%	2.16%	2.65%	1.19%	0.66%
(Z)-4-Decen-1-ol	41.74	156	0.00%	0.16%	0.16%	0.07%	0.04%
Phenol	44.99	94	0.03%	0.03%	0.05%	0.03%	0.01%
cis-2,3-Epoxycyclohexane-1-methanol	46.14	128	0.08%	0.00%	0.06%	0.05%	0.01%
Benzyl alcohol	46.2	108	0.03%	0.00%	0.00%	0.00%	0.00%
Phenol, 2-methoxy-4-(2-propenyl)	49.74	164	0.08%	0.01%	0.03%	0.01%	0.00%
Eugenol	50.46	164	0.03%	0.00%	0.00%	0.00%	0.00%
**Aldehydes**			**14.44%**	**2.71%**	**1.75%**	**1.01%**	**0.86%**
2-Methylbutyraldehyde	8.63	86	0.07%	0.88%	0.17%	0.01%	0.00%
Hexanal	13.09	100	0.35%	0.22%	0.16%	0.05%	0.06%
2-Pentenal,(E)-	14.32	84	0.59%	0.09%	0.10%	0.03%	0.04%
2-Hexenal, (E)-	16.39	98	1.91%	0.11%	0.16%	0.06%	0.04%
2,4-Heptadienal	24.79	110	4.18%	0.55%	0.24%	0.33%	0.52%
2,6-Nonadienal, (E,Z)	28.33	138	2.08%	0.08%	0.09%	0.08%	0.07%
1-Cyclohexene-1-carboxaldehyde, 2,6,6-trimet	30.08	152	0.62%	0.34%	0.18%	0.10%	0.04%
Benzeneacetaldehyde	31.02	120	3.77%	0.43%	0.62%	0.31%	0.07%
4-Heptenal,(Z)	32.27	112	0.41%	0.00%	0.00%	0.00%	0.00%
2,4-Decadienal,(E,E)	38.19	152	0.18%	0.02%	0.04%	0.04%	0.01%
3-Cyclohex-1-enyl-prop-	41.45	139	0.30%	0.00%	0.00%	0.00%	0.00%

2-enal-1							
**Alkanes**			**0.37%**	**10.03%**	**6.95%**	**0.71%**	**0.79%**
2,2-Dimethyldecane	9.34	170	0.03%	0.92%	0.69%	0.09%	0.10%
Decane	10.59	142	0.10%	4.20%	1.96%	0.18%	0.22%
Octane, 3,3-dimethyl-	10.79	142	0.03%	1.21%	0.70%	0.07%	0.08%
Heptane, 3,3,5-trimethyl-	10.97	100	0.06%	1.10%	1.22%	0.13%	0.16%
Dodecane	15.38	170	0.14%	2.60%	2.38%	0.24%	0.23%
**Alkenes**			**0.15%**	**0.09%**	**1.16%**	**0.91%**	**0.99%**
α-PINENE	11.44	136	0.06%	0.03%	0.71%	0.47%	0.39%
trans-Caryophyllene	29.01	204	0.05%	0.03%	0.20%	0.17%	0.23%
α-Amorphene	32.81	204	0.02%	0.01%	0.12%	0.13%	0.17%
δ-Cadinene	35.84	204	0.02%	0.02%	0.12%	0.14%	0.20%
**Aromatic hydrocarbons**			**0.03%**	**3.56%**	**0.09%**	**0.03%**	**0.04%**
p-Xylene	14.59	106	0.03%	1.61%	0.07%	0.01%	0.03%
Naphthalene, 1,2,3,4-tetrahydro-1,1,6-trimethy	33.14	174	0.00%	0.44%	0.01%	0.01%	0.01%
Naphthalene, 1,2-dihydro-1,1,6-trimethyl-	35.65	172	0.00%	1.51%	0.02%	0.00%	0.00%
**Esters**			**2.89%**	**1.99%**	**1.61%**	**1.06%**	**1.12%**
Butyrolactone	30.76	86	0.04%	0.57%	0.30%	0.02%	0.00%
Benzoic acid, 2-hydroxy-,methyl ester	37.1	174	0.50%	0.47%	0.34%	0.33%	0.48%
Nonanoic acid, 9-oxo-, methyl ester	46.48	186	0.28%	0.03%	0.05%	0.00%	0.00%
Methyl 13-methyltetradecanoate	47.87	256	0.03%	0.01%	0.02%	0.00%	0.00%
Hexadecanoic acid, methyl ester	50.66	270	1.73%	0.35%	0.33%	0.25%	0.24%
Methyl 8,11,14-heptadecatrienoate	59.59	278	0.31%	0.56%	0.56%	0.45%	0.40%
**Ketones**			**4.78%**	**0.94%**	**0.51%**	**0.29%**	**0.29%**
1-Penten-3-one	11.56	84	2.30%	0.21%	0.14%	0.05%	0.05%
4-Methyl-2-heptanone	18.35	128	0.18%	0.00%	0.00%	0.00%	0.00%
3,5-Octadien-2-one	25.58	124	0.76%	0.17%	0.04%	0.03%	0.03%
3-Nonen-2-one	29.02	140	0.00%	0.00%	0.04%	0.04%	0.05%
2,5-Dimethyl-4-hydroxy-3(2H)-furanone	40.09	128	0.00%	0.28%	0.14%	0.00%	0.00%
3-Buten-2-one, 4-(2,2,6-trimethyl-7-oxabicyclo[4.1.0]hept-1-yl)-	44.75	208	1.54%	0.27%	0.16%	0.17%	0.16%
**Organic acids**			**2.01%**	**0.65%**	**2.26%**	**0.91%**	**0.22%**
Hexanoic acid	39.37	116	0.86%	0.24%	0.60%	0.56%	0.19%
3-Hexenoic acid, (E)-	43.64	114	0.26%	0.07%	0.43%	0.07%	0.00%
2-Hexenoic acid	43.91	114	0.67%	0.16%	1.06%	0.20%	0.00%
Octanoic acid	46.51	144	0.22%	0.17%	0.17%	0.08%	0.03%
**Others**			**3.31%**	**4.32%**	**3.41%**	**2.47%**	**3.08%**
(3R)-3-Phenyl-2,3-dihydro-1H-isoindol-1-one	6.5	251	1.96%	3.76%	2.77%	2.00%	1.87%
1-Butene, 2-(chloromethyl)-	11.21	105	0.47%	0.14%	0.19%	0.13%	0.17%
N-benzylidene-	26.21	340	0.38%	0.36%	0.34%	0.24%	1.03%

dimethylammonium chloride							
1H-Pyrrole-2,5-dione,3-ethyl-4-methyl	52.36	139	0.50%	0.06%	0.12%	0.09%	0.02%

The data are as presented in percentage for each volatile under total peak area of fresh conditions. Values in bold type are the totals within each compound group.
